# Age-Related Differences in the Effects of Masker Cuing on Releasing Chinese Speech From Informational Masking

**DOI:** 10.3389/fpsyg.2018.01922

**Published:** 2018-10-09

**Authors:** Tianquan Feng, Qingrong Chen, Zhongdang Xiao

**Affiliations:** ^1^College of Teacher Education, Nanjing Normal University, Nanjing, China; ^2^State Key Laboratory of Bioelectronics, School of Biological Science and Medical Engineering, Southeast University, Nanjing, China; ^3^School of Psychology, Nanjing Normal University, Nanjing, China

**Keywords:** speech recognition, informational masking, masker cuing, age effects, voice

## Abstract

The aims of the present study were to examine whether familiarity with a masker improves word recognition in speech masking situations and whether there are age-related differences in the effects of masker cuing. Thirty-two older listeners (range = 59–74; mean age = 66.41 years) with high-frequency hearing loss and 32 younger normal-hearing listeners (range = 21–28; mean age = 23.73) participated in this study, all of whom spoke Chinese as their first language. Two experiments were conducted and 16 younger and 16 older listeners were used in each experiment. The masking speech with different content from target speech with syntactically correct but semantically meaningless was a continuous recording of meaningless Chinese sentences spoken by two talkers. The masker level was adjusted to produce signal-to-masker ratios of -12, -8, -4, and 0 dB for the younger participants and -8, -4, 0, and 4 dB for the older participants. Under masker-priming conditions, a priming sentence, spoken by the masker talkers, was presented in quiet three times before a target sentence was presented together with a masker sentence 4 s later. In Experiment 1, using same-sentence masker-priming (identical to the masker sentence), the masker-priming improved the identification of the target sentence for both age groups compared to when no priming was provided. However, the amount of masking release was less in the older adults than in the younger adults. In Experiment 2, two kinds of primes were considered: same-sentence masker-priming, and different-sentence masker-priming (different from the masker sentence in content for each keyword). The results of Experiment 2 showed that both kinds of primes improved the identification of the targets for both age groups. However, the release from speech masking in both priming conditions was less in the older adults than in the younger adults, and the release from speech masking in both age groups was greater with same-sentence masker-priming than with different-sentence masker-priming. These results suggest that both the voice and content cues of a masker could be used to release target speech from maskers in noisy listening conditions. Furthermore, there was an age-related decline in masker-priming-induced release from speech masking.

## Introduction

In everyday scenarios, individuals are often faced with the difficulty of interpreting the speech of one person while other people are speaking simultaneously (Cherry, 1953). In noisy listening environments, two main factors are thought to contribute to this difficulty: energetic masking (Leek et al., 1991) and informational masking (Freyman et al., 1999; Arbogast et al., 2002; Kidd et al., 2005; Schneider et al., 2007; Agus et al., 2009; Helfer and Freyman, 2009). Energetic masking occurs at the auditory periphery, when components of the speech signal in some time–frequency region are rendered inaudible because of swamping by the masker (Leek et al., 1991; Kidd et al., 1994, 1998; Freyman et al., 1999), such that the response of the peripheral neurons to the target is suppressed by that evoked by the masker. In contrast to energetic masking, informational masking is a kind of higher-level masking beyond the periphery and exerts its influence at a more cognitive level, making it difficult to identify and attend to the target. Therefore, the contributions of the different types of masking to speech identification are different and depend on the nature of the distractor sound sources. Steady noise is considered to produce primarily energetic masking [even though Stone et al. (2011, 2012) recently demonstrated that the inherent random amplitude fluctuations of such a masker also impede speech identification, presumably due to modulation masking], while a speech masker produces both energetic and informational masking.

To improve target speech recognition in noisy environments, available perceptual/cognitive cues can be used to orient selective attention (Best et al., 2007) toward the target or to inhibit the effects of non-target speech signals. If perceptual/cognitive cues are available that contribute to the selective attention of listeners to target words or sentences and neglecting of distracting sentences, target sentence identification can be improved by reducing the speech-masker-induced masking (Leek et al., 1991; Kidd et al., 1994, 1998, 2005; Freyman et al., 1999, 2001; Arbogast et al., 2002; Schneider et al., 2007; Wu et al., 2007; Agus et al., 2009; Helfer and Freyman, 2009). The degree of informational masking is closely correlated with the similarity between the voice characteristics of the target and masker speakers (i.e., their genders) (Brungart et al., 2001). It has been demonstrated that the influence of informational masking can be reduced by providing certain perceptual cues that can help subjects perceptually extract target sentence information from speech distractors (Freyman et al., 2004). Forewarning of the nature of the target speech (called the “priming effect”) familiarizes the listeners with the voice characteristics, content, and location of the target, allowing them to attend to the target speech selectively and improving their recognition of the target sentence (Sumby and Pollack, 1954; Summerfield, 1979; Rosenblum et al., 1996; Grant and Seitz, 2000; Brungart et al., 2001; Rudmann et al., 2003; Freyman et al., 2004; Kidd et al., 2005; Helfer and Freyman, 2005, 2008; Rakerd et al., 2006; Newman and Evers, 2007). It should be noted that the degree of informational masking can also be reduced if an early part of a target sentence (called a “content prime”) is presented before the full target sentence. Furthermore, presentation of a prime via a different medium, e.g., via a different target-speaker voice (male or female) or in writing, has been shown to improve performance compared to that without priming and with the same degree of informational masking (Freyman et al., 2004). In other words, priming is efficient provided that the prime can help listeners attend to the target within the masking background. Different effects can also be noted for different languages. In regards to this study, it should be emphasized that Chinese speech is quite different from English speech, in terms of both pronunciation and sentence structure, which may yield a different informational masking effect. Following replication of and expansion upon previous work (Freyman et al., 1999) and using Mandarin-speaking Chinese subjects, the priming effects in Chinese speech-on-speech masking scenarios also have been studied (Wu et al., 2005, 2012a,b; Yang et al., 2007).

Notably, older listeners often experience difficulty understanding speech under noisy listening conditions (Duquesnoy, 1983; Gelfand et al., 1988; Helfer and Wilber, 1990; Humes and Roberts, 1990; Jerger et al., 1991; Cheesman et al., 1995; Dubno and Ahlstrom, 1997; Frisina and Frisina, 1997; Yonan and Sommers, 2000; Schneider et al., 2000; Tun et al., 2002; Rossi-Katz and Arehart, 2009; Helfer et al., 2010; Huang et al., 2010; Demeester, 2011). It was also shown that the ability to identify target speech in a masker declines in older adults even if they have normal hearing sensitivity and are audiometrically matched to younger control listeners (Füllgrabe et al., 2015). The existing reports attribute these age-related difficulties to age-related cognitive declines and auditory processing deficits. Age-relative cognitive declines are manifested in attention, working memory, inhibitory control, and processing speed under noisy listening conditions (Schneider, 1997; Schneider et al., 2007) and may also be correlated with speech-perception performance (Füllgrabe et al., 2015). Age-related auditory processing deficits (specifically, audiometric losses and supra-threshold auditory processing deficits) may be responsible for these difficulties (Jerger, 1992; Schneider, 1997; Füllgrabe et al., 2003, 2015; Kricos, 2006; Schneider et al., 2007; Moore et al., 2012; Füllgrabe, 2013; Wallaert et al., 2016; Lopez-Poveda et al., 2017). These deficits can have different origins, such as hearing loss (Hutka et al., 2013; Füllgrabe and Moore, 2014) and hearing-sensitivity-independent age-related changes (Füllgrabe et al., 2015; Musiek et al., 2017), among others. The existing studies have also shown that sensory deterioration in older listeners and resulting degeneration of acoustic information (Huang et al., 2008) could result in decreasing stream segregation efficiency (Huang et al., 2009; Li et al., 2009) due to hearing loss (Hutka et al., 2013; Füllgrabe and Moore, 2014) and thus a lesser degree of target speech release from informational masking. It may also be that older listeners have to redistribute their cognitive resources to compensate for poor sensory input, thus consuming the resources available for language processing (Schneider, 1997; Schneider et al., 2007). However, one investigation (Ezzatian et al., 2011) demonstrated that the benefits of priming are equivalent among both older and younger English-speaking adults (with normal audiometric thresholds, i.e., less than 25 dB HL up to and including 4 kHz), indicating that both groups can use primes to facilitate auditory scene analysis and word recognition. By replicating and expanding upon these investigations (Ezzatian et al., 2011) using Mandarin-speaking Chinese listeners (with mismatched audiometric thresholds between younger and older listeners), the influences of age on the ability to obtain prior knowledge about information in speech identification under masking have been investigated (Wu et al., 2012b). Older listeners experience more difficulty than younger listeners in following and comprehending spoken language in complex acoustic scenes since the ability to benefit from cues could be compromised by both cognitive and auditory declines in older listeners. However, the existing studies did not specify the exact contributions of these factors.

Most previous studies have focused on the influence of target cues on extracting target speech from informational masking. Those studies have shown that target cues can help listeners attend targets and effectively improve target recognition performance. However, background masking is another important factor in identifying target speech in complex acoustic environments, which raises the question of whether background masking cues can help the auditory systems of listeners extract targets from such mixtures effectively. In this regard, it should be noted that the effects of masker cuing have been studied previously in auditory enhancement experiments (Viemeister, 1980; Viemeister and Bacon, 1982; Byrne et al., 2011), which demonstrated that prior exposure to a harmonic complex lacking a pure-tone component could markedly improve the recognition of the missing pure tone when the full harmonic complex was presented. This finding implies that cuing to the masker (the harmonic complex without the pure-tone component) could improve the ability of the listeners to recognize the target signal (the pure-tone component). Those studies indicated that the decreased effectiveness of masking results from simple adaptation to its frequency components, which occurs when listeners hear a masker beforehand (Byrne et al., 2011). In addition to the auditory enhancement effect for pure-tone identification, the effects of masking speech familiarity on young adults in speech-on-speech masking have also been studied recently (Zhang et al., 2012), revealing that prior knowledge of the masker enhances the informational masking and renders target recognition more difficult in young adults.

In this paper, we address whether prior familiarity with a masker affects word recognition in speech masking situations among younger and older adults and whether there are age-related differences in the abilities of listeners to utilize masking speech cues for target speech identification. The masker prime was the same as the masking speech, which was presented before a Chinese target sentence masked by two-talker speech. The targets were Chinese nonsense sentences that are syntactically correct. The two-talker speech masker sentences consisted of the same kind of syntactically correct but semantically meaningless sentences. Two kinds of masker primes were investigated: the same sentence as the masker (same-sentence masker-priming) and a sentence different from the masker (different-sentence masker-priming). In the masker-priming conditions, the presentation of each masker-priming sentence was repeated three times so that the listeners could become familiar with the masker cues. The performance in each situation was compared to that without priming. It should be noted that the ability to perceive and remember speech is decreased in older adults. Different-sentence masker-priming was utilized to examine whether masker voice cues, masker content cues, or these two factors combined affect younger and older adults in target sentence word identification. The performance in these different masker-priming conditions was evaluated separately for each of three keywords in the target sentence, and then for the whole target sentence.

## Experiment 1: Same-Sentence Masker-Priming

In Experiment 1, we examined whether familiar masking cues can enhance word identification performance in speech masking situations among younger and older adults and whether younger and older adults differ in their abilities to utilize such masker-priming.

### Materials and Methods

#### Participants

Sixteen Mandarin Chinese-speaking younger participants (nine females and seven males), and 16 Mandarin Chinese-speaking older participants (ten females and six males) participated in the experiments. The mean ages of the younger and older participants were 24 years (range: 22 - 28 years) and 65 years (range: 59 - 73 years), respectively. The younger adults were graduate students recruited from Nanjing Normal University. The older adults were recruited from the communities of Nanjing Normal University and Southeast University teachers who were retired trade union members of the two universities. All of the older participants completed the Chinese version of the Montreal Cognitive Assessment test to screen for cognitive impairment (more than 26/30 points was designated as exhibiting no cognitive impairment), and all of them obtained full marks. In addition, most of the older participants and all of the younger participants had completed undergraduate education, and none of the participants suffered from cognitive impairment.

**FIGURE 1:**
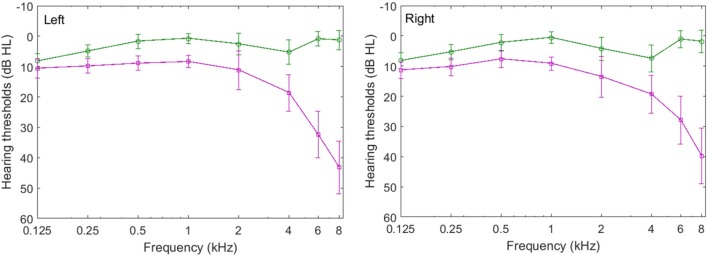
Average audiometric thresholds in the left ear **(left)** and right ear **(right)** for all younger (circles) and older participants (squares) who participated in Experiment 1. The error bars represent the standard deviations of the means.

Pure-tone air-conduction audiometry was conducted by using an ITERA Clinical Audiometer with TDH-39 headphones for all of the participants, following the procedure recommended by the Standardization Administration of the People’s Republic of China (GB/T 16403-1996). In the present study, the normal audiogram range was defined as an audiometric threshold less than 25 dB HL at test frequencies of 0.125, 0.25, 0.5, 1, 2, 4, 6, and 8 kHz. The average audiometric thresholds of the younger and older participants are summarized in **Figure 1**, which shows that these thresholds for the younger and older participants were similar below 4 kHz in both the left and right ears. On average, the audiometric thresholds of the older adults are about 8 dB higher than those of the younger listeners up to and including 2 kHz. However, the age-related audiometric threshold difference increased for frequencies above 2 kHz, and was 13 dB at 4 kHz. Particularly, the difference between the audiometric thresholds of the younger and older participants was greater than 30 dB HL but no more than 65 dB HL at frequencies of 6 and 8 kHz. An age group by frequency analysis of variance (ANOVA) of the average audiometric thresholds for all eight frequencies (0.125–8 kHz) showed that the group (*F*_1,30_ = 8.177, *p* = 0.003) and audiometric frequency (*F*_7,210_ = 5.661, *p* = 0.012) had significant main effects, and that there was a significant interaction between age group and audiometric frequency (*F*_7,210_ = 3.753, *p* = 0.031). These results indicate that hearing sensitivity of the participants in the two groups differed. Two of the older adults had audiometric thresholds higher than 25 dB HL at frequency 2 kHz. On average, older adults had clinically normal hearing in both ears from 0.125 to 2 kHz, but they were likely experiencing clinical declines for frequencies above 2 kHz. In the present study, the bandwidth of the speech signals was filtered between 0.125 and 8 kHz, and was not limited to the audiometric normal range. In addition, the interaural difference was less than 15 dB (at each frequency) for the younger and older groups. These results indicate that all of the participants had symmetrical hearing between the left and right ears.

All of the participants provided informed consent to join the present study, which was conducted in accordance with the Declaration of Helsinki and was approved by the Ethics Committee of the Nanjing Normal University.

Each participant sat on a stool with back in the center of a silent room. The length, width, and height of the room were, respectively, 350, 240, and 250 cm. The sound signals were recorded digitally on a computer with an i7 CPU (Intel Core, Santa Clara, CA, United States), digitalized at a 22.05 kHz sampling rate using a 24-bit Creative Sound Blaster PCI128 (Creative Technology Co., Ltd., Singapore), and edited using Cooledit Pro 2.1 (Syntrillium Software Corporation, Phoenix, AZ, United States). A Dynaudio Acoustics loudspeaker (Dynaudio, Risskov, Denmark) was located at a height of 100 cm in front of the participants. The distance between the head of each participant and the loudspeaker was 160 cm.

#### Experimental Stimuli and Apparatus

The speech stimuli were meaningless Chinese sentences with correct syntactical structures, but incongruous semantics. The direct English translations of these Chinese sentences have syntax similar to but not exactly the same as that of the meaningless English sentences in other previous studies (Helfer, 1997; Freyman et al., 1999, 2004; Ezzatian et al., 2011). Each of the Chinese nonsense sentences had a subject (noun)–predicate (verb)–object (noun) structure and provided no contextual support for keyword recognition (Wu et al., 2005, 2011, 2012a,b; Yang et al., 2007). Each of the three parts of the Chinese nonsense sentences, i.e., the subject, predicate, and object, had two characters (syllables). In this study, the subject, predicate, and object keywords represented, respectively, the initial, middle, and final keywords. Each keyword in the target sentence was scored separately (as correct or incorrect) during the speech identification testing. To ensure that these sentences were meaningless, the probability of two nouns and a verb co-occurring in each sentence was determined based on the total vocabulary data of the Chinese magazine ***Readers*** over 6 years (2005 - 2010). For example, one of the meaningless Chinese sentences could be translated word-for-word into English as “His **teeth** will **like** that **potato**” (the three keywords appear in bold font).

In this work, a considerable amount of meaningless sentence stimuli was needed. All of the target sentences were spoken by three young Chinese females (speakers A, B, and C) in Standard Mandarin. The masking speech was a continuous recording of meaningless Chinese masking sentences (which did not contain the keywords in the targets) synthesized artificially by two other young Chinese females (speakers D and E), who spoke the same sentences. The evident pauses in the masking speech signal were removed to obtain a continuous stream without gaps, thereby reducing the possibility of the participants hearing the target sentence clearly during the gaps in the masking speech. The target sentence was presented in such a masking background composed of two interfering speakers during the target/masker experiments. All of the speech signal were calibrated utilizing a Type 2230 B & K sound-level meter, whose microphone was located at a position corresponding to the center of the head of a participant (this calibration was conducted in absence of the participants), using the root-mean-square meter response. To overcome the influences of the floor and ceiling, the targets were presented with a fixed level (60 dBA). The different signal-to-masker ratios (SMRs) could be obtained by varying the masker pressure (Ezzatian et al., 2011).

#### Experimental Design and Procedure

For each group of participants, there were eight experimental conditions, which were obtained by crossing two prime conditions with four SMRs. Sixteen lists of meaningless Chinese sentences were used as the target sentences. Each list contained 18 target sentences. The participants were informed of the type of priming condition (with or without priming) during a test session. The SMRs remained constant throughout the presentation of a single list, and the four different SMRs were arranged randomly across the lists. The sentence lists and SMRs were counterbalanced across the participants such that each list was presented at each of the four different SMRs. The masker pressure was adjusted to produce SMRs of -12, -8, -4, and 0 dB for the younger participants. For the older participants, the masker pressure was adjusted to produce another four SMRs to minimize the floor effect: -8, -4, 0, and 4 dB. A study by Li et al. (2004) showed that, compared to the younger participants, the older participants required a 2.8 dB higher SMR to achieve the same level of accuracy.

To balance the information quantity in the various experimental scenarios, the information quantity of a keyword in a single sentence was defined as follows:

$$Q=-\log(\frac{1}{\omega_{f}}),$$

with *ω_f_* being the frequency of the word. The *Q* value for each sentence was the sum of its values for all of the keywords. All of the sentences in each list were chosen such that the value of each list was roughly constant.

In each experimental trial, each participant pressed a button on a keyboard to start. In the case of no priming, the speech stimuli were presented according to the following procedure: (1) the presentation of a two-talker masker began immediately after the button press; (2) a full target sentence was presented about 500 ms after the masker onset; (3) the masker and target ended simultaneously. With same-sentence masker-priming, the speech stimuli were presented according to four temporal stages: (1) the prime (which was the same as the full masker sentence) was presented three times in silence immediately after the button press, and there was no pause between the three presentations of the prime; (2) the presentation of a masker sentence (produced by two masker voices) began about 4000 ms after the end of the prime presentation; (3) a full target sentence was presented about 500 ms after the masker onset; (4) the masker and target ended simultaneously. Note that the three presentations of the masker-priming sentence were intended to familiarize the participants with the masker, and the 4000 ms interval between the prime and masker was to prepare the participants for the following identification task and increase their attention to the target.

The task of each participant was to repeat the full target sentence orally as accurately as possible after the end of each presentation. One person seated outside the anechoic chamber scored the performance of the participant. The three keywords in the Chinese target sentence were scored separately. Because each keyword had two characters (syllables), the performance was scored as the number of correctly identified characters for each keyword. The percentage correct for each keyword was the ratio of the number of characters identified correctly to the total number of characters in each keyword. The number of correctly identified characters for the whole target sentence (three keywords) was tallied later. The percentage correct for the whole target sentence was the ratio of the number of characters identified correctly to the total number of characters in the whole sentence.

Before the formal experiments, six Mandarin Chinese-speaking older participants (three females and three males) with a mean age of 65.1 years (range: 55–74 years) were invited to evaluate the number of exposures (two or three) and the interval (3000 or 4000 ms) between the masker prime and target/masker presentation under different-sentence masker-priming conditions. In different-sentence masker-priming condition, the prime sentence were spoken by the masker talkers so that the voices were the same for the prime and corresponding masker sentences; however, none of the words in the primes were related to the words in the masker sentences. The results showed that the average value of the threshold μ (50% correct threshold) for identification of the whole target sentence was lower when the masker prime was presented three times than when it was presented two times, regardless of whether the delay between the masker prime and the speech presented in the masker was 3000 ms (-1.0 dB vs. -0.7 dB) or 4000 ms (-1.1 dB vs. -0.9 dB) under different-sentence masker-priming conditions. These results were confirmed by performing an ANOVA, which showed that the main effect of the number of exposures was significant (*F*_1,5_ = 5.912, *p* = 0.029), that the main effect of the delay was not significant (*F*_1,5_ = 2.224, *p* = 0.146), and that there was a significant interaction between the number of exposures and delay (*F*_1,5_ = 4.750, *p* = 0.037).

To ensure that all of the participants understood the task and the instructions, a training session was conducted prior to data collection. A visually aided explanation of the instructions was presented to clarify the experimental procedures mentioned above to each participant. To familiarize the participants with the task, each participant also performed a test to identify one of the practice sentences with a high SNR both with and without priming.

### Results

**FIGURE 2:**
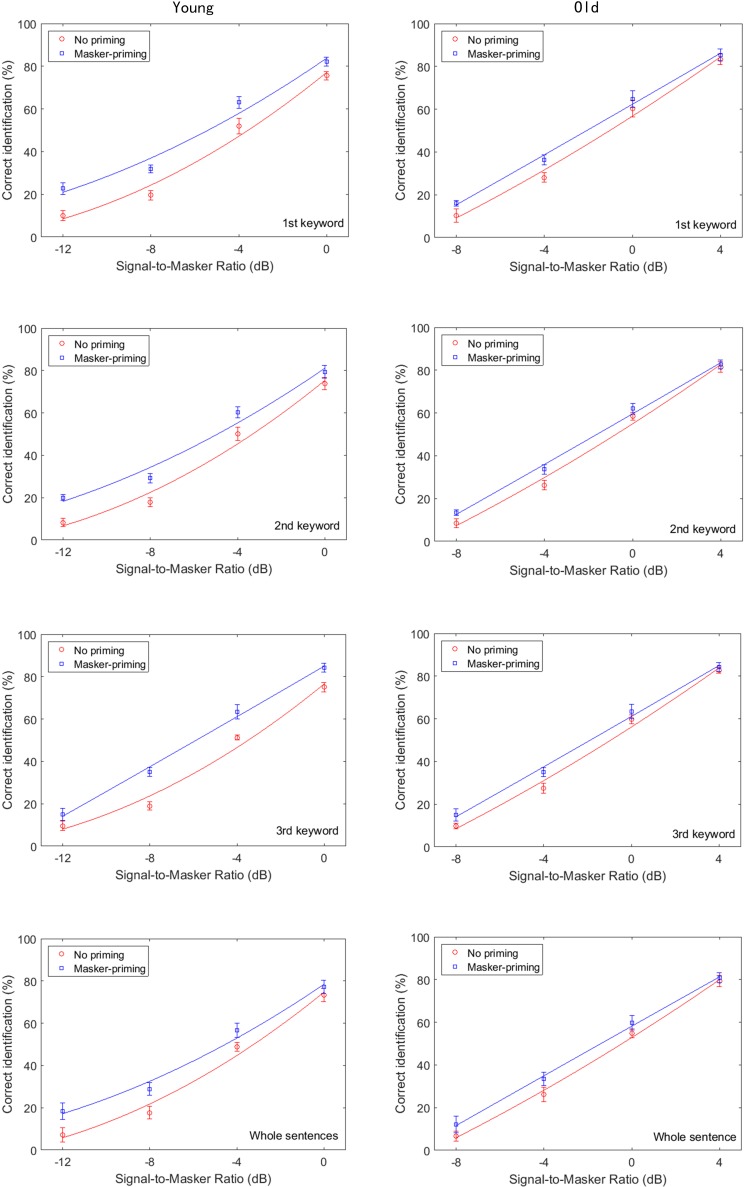
Group-mean percentages of correct identification of each of the three keywords in the target sentence in Experiment 1, as a function of signal-to-masker ratio (SMR) and for the younger (left) and older participants (right). The **top, upper middle, lower middle, and bottom panels**, respectively, represent the group-mean percentages of correct identification of the initial keyword, middle keyword, final keyword, and whole target sentence. Two different priming conditions were implemented: no priming (circles) and same-sentence masker-priming (squares). The best-fit psychometric functions (curves) for each masker-priming condition are shown in each of the panels.

This section presents a statistical analysis of the measured effects of masker-priming on the target speech recognition by the younger and older subjects. **Figure 2** displays the group-mean percentages for correct identification of each keyword in the target sentence and the whole sentence as a function of the SMR for the younger (left) and older participants (right). The results are shown for the cases in which no priming was presented (circles) and same-sentence masker-priming was presented (squares). The smooth lines correspond to the psychometric function

$$p(y)=\frac{1}{1+\exp\left[-\sigma \left(x-\mu \right)\right]},$$

where *y* is the probability of correct keyword identification in the target sentences for different SNRs, *x*, and μ and σ are the SMR for 50% correctness and the slope, respectively. The values of μ and σ that were used to generate the curves in **Figure 2** (solid lines) were those that minimized Pearson’s χ^2^ goodness of fit of the model to the data of each participant (see Yang et al., 2007 for a description of the fitting procedure). As shown in **Figure 2**, higher group-mean percentages of correct identification for each of the three keywords and the whole sentence were obtained with masker-priming than without priming by both the younger and older adults. An examination of **Figure 2** suggests that the amount of release from the speech masking induced by the masker-priming was slightly greater for the younger adults than for the older adults.

**FIGURE 3:**
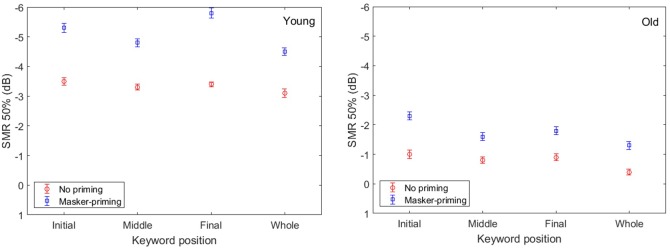
Average threshold value (μ) as a function of keyword position in Experiment 1, for the younger and older participants and with and without masker-priming. The blue and red rectangles indicate the data obtained with and without masker-priming, respectively. The **(left and right)** respectively, correspond to the average μ for the younger and older participants. The error bars indicate the standard errors of the means.

To determine whether the psychometric functions shown in **Figure 2** also characterized the individual participants, we fit individual psychometric functions to the data from each individual. The average values of μ for both the younger and older participants for identification of each keyword and the whole sentence with and without masker-priming are displayed in **Figure 3**. The threshold values are evidently lower for the younger adults than for the older adults for each keyword in both masker-priming conditions, which is reasonable because the older participants required about a 3 dB higher SMR to perform at the same level as the younger participants (Ezzatian et al., 2011). The release of each keyword in the target sentence due to masker-priming was greater for the younger adults (1.8, 1.5, and 2.4 dB for the initial, middle, and final keywords, respectively) than for the older adults (1.3, 0.8, and 0.9 dB for the initial, middle, and final keywords, respectively). For the whole sentence, the amount of release resulting from masker-priming was greater for the younger adults than for the older adults (1.4 dB vs. 0.9 dB). Although the average audiometric thresholds in **Figure 1** show that the older participants had normal hearing sensitivity up to 4 kHz but a mild-to-moderate hearing loss at higher frequencies. It should be noted that the cutoff frequency of the speech stimuli used in this study was 8 kHz and hence performance in the older adults might have been worse compared to that in younger adults due to their high-frequency loss. Finally, the amount of release from speech masking induced by masker-priming was greater for the final keyword (2.4 dB) than for the other two keywords (1.8 and 1.5 dB for the initial and middle keywords, respectively) among the younger adults. However, among the older participants, the amount of release from speech masking due to masker-priming was greater for the initial keyword (1.3 dB) than for the other keywords (0.8 and 0.9 dB for the middle and final keywords, respectively).

An ANOVA of the threshold values with age group as a between-subjects factor and keyword position and priming condition as within-subject factors demonstrated that there were highly significant effects due to age (*F*_1,30_ = 151.076, *p* < 0.001) and priming condition (*F*_1,30_ = 45.193, *p* < 0.001). However, the main effect of the keyword position on the threshold value was not significant (*F*_2,60_ < 1). In addition, the interactions between age and priming condition (*F*_1,30_ < 1) and between keyword position and age (*F*_2,60_ = 2.047, *p* = 0.141) were not significant. However, the interaction between keyword position and priming condition was significant (*F*_1,30_ = 24.259, *p* < 0.01), and the three-way interaction among age, keyword position, and priming condition was highly significant (*F*_2,60_ = 62.164, *p* < 0.001).

The ANOVAs with keyword position and priming condition as within-subject factors were conducted separately for the younger and older participants to confirm the source of this three-way interaction. For the younger participants, the ANOVA revealed that the main effects of both priming condition (*F*_1,15_ = 34.224, *p* < 0.01) and keyword position were significant (*F*_2,30_ = 16.417, *p* < 0.01) and that the interaction between keyword position and priming condition was significant (*F*_2,30_ = 22.168, *p* < 0.01). Therefore, for the younger participants, the effect of the priming condition differed among the three keywords. Multiple *t*-tests (Bonferroni corrected, the results of all subsequent *t*-tests were corrected for multiple comparisons) confirmed that there were significant differences between the scenarios with and without priming for the initial (*t*_15_ = 6.933, *p* < 0.01), middle (*t*_15_ = 7.045, *p* < 0.01), and final keywords (*t*_15_ = 12.641, *p* < 0.01).

For the older participants, the equivalent ANOVAs with keyword position and priming condition as within-subject factors revealed that both priming condition (*F*_1,15_ = 19.081, *p* < 0.01) and keyword position (*F*_2,30_ = 11.172, *p* < 0.01) were significant, as was the interaction between keyword position and priming condition (*F*_2,30_ = 14.066, *p* < 0.01), which implies that the effects of masker-priming also differed among the three keywords. The multiple *t*-tests confirmed that there were also significant differences between the situations with and without masker-priming for the initial (*t*_15_ = 19.371, *p* > 0.05), middle (*t*_15_ = 10.114, *p* < 0.01), and final keywords (*t*_15_ = 13.135, *p* < 0.01).

For the whole target sentences, a two-factor ANOVA showed that the main effects of both age (*F*_1,30_ = 76.066, *p* < 0.01) and priming condition (*F*_1,30_ = 14.066, *p* < 0.01) were significant, but the interaction between age and priming condition was not significant (*F*_1,30_ < 1). The pairwise *t*-tests showed that there was a significant difference between the situations with and without masker-priming (*t*_15_ = 4.984, *p* < 0.01). Therefore, the thresholds were higher for the older participants than for the younger participants, and the thresholds were higher without priming, which implies that the masker-priming led to the release from the masking.

### Discussion

The results of Experiment 1 reveal for the first time that presenting the whole masking sentences in silence before presenting the target/masker significantly improves the identification of each keyword in the whole target sentences among both younger and older participants. The present findings could be interpreted as indicating that the repetitive presentation of the masking speech (masker-priming) familiarizes listeners with it and makes them more sensitive to the target speech in the target/masker presentation. Perhaps this feature is a kind of adaptation or repetitive inhibitory effect of the human central auditory system, leading to neural activity reduction (Ulanovsky et al., 2003; Perez-Gonzalez et al., 2005; Grill-Spector et al., 2006). The presentation of target speech arouses the auditory nerve system and orients the attention of the listeners to the targets, leading to the release of the target speech from the familiar masker.

In addition, the results of this experiment demonstrate that the amount of release from speech masking induced by masker-priming was greater for the younger participants than for the older participants. One possible explanation of this finding could be that the hearing sensitivity of the older listeners was worse than that of the younger participants. Such age-related audiometric deficits may have prevented the older listeners from benefiting from masker-priming during the target/masker presentation. Alternatively, the less amount of masking release among the older participants may have resulted from suprathreshold auditory deficits (e.g., reduced frequency selectivity) associated with high-frequency hearing loss and aging. It has been shown that age-related audiometric deficits in speech perception (Moore, 2007; Hopkins and Moore, 2011) are related to the hearing loss at high frequencies. Such audiometric deficits have deleterious effects on speech perception even with very minor hearing losses (30 dB or less). Furthermore, the results of the present study indicate that the effects of masker-priming differed among the three keywords in both age groups.

## Experiment 2: Same-Sentence Masker-Priming Vs. Different-Sentence Masker-Priming

Since both the content and voice cues of the masker provided by masker-priming could independently or in combination have led to improved identification of target speech in the presence of masker speech in Experiment 1, the effect of two primes (same-sentence masker-priming and different-sentence masker-priming) was investigated in Experiment 2. The same-sentence masker-priming provided the listeners with the voice and content cues of the masker simultaneously, and the different-sentence masker-priming provided the listeners with only the voice cue of the masker talker. In this experiment, we examined whether the familiar voice cue of the masker alone led to release from speech masking and whether there were differences between the release from speech masking using the two kinds of masker-priming conditions. In Experiment 2, in addition to examining which of the factors led to target speech identification improvement, we investigated whether there were any age-related differences.

### Materials and Methods

#### Experimental Participants

Sixteen Mandarin Chinese-speaking younger participants, including nine females and seven males, and 16 Mandarin Chinese-speaking older adults, including 10 females and six males, participated in the experiments. The mean ages of the younger and older participants were 23 years (range: 21 - 26 years) and 68 years (range: 62 - 74 years), respectively. All of the participants met the same criteria as those described for Experiment 1, but they did not participate in Experiment 1.

**FIGURE 4:**
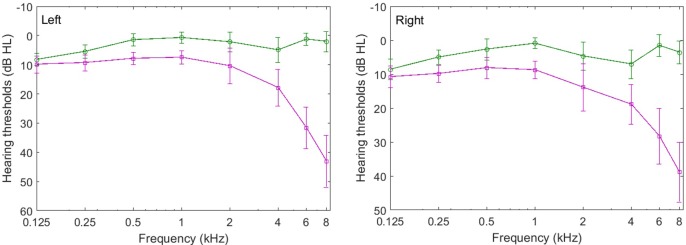
Average audiometric thresholds in the left ear **(left)** and right ear **(right)** for all younger (circles) and older participants (squares) who participated in Experiment 2. The error bars represent the standard deviations of the means.

The average audiometric thresholds of the younger and older participants in this experiment are shown in **Figure 4**, where it can be seen that average audiometric thresholds of the younger and older participants are similar below 4 kHz in both left and right ears. On average, the audiometric thresholds are about 7 dB higher for the older listeners than for the younger listeners up to and including 2 kHz. However, the age-related audiometric threshold difference increased with frequency for frequencies above 2 kHz, and was higher than 12 dB at frequency 4 kHz. Particularly, the difference between the audiometric thresholds of the younger and older participants is greater than 30 dB HL but no more than 65 dB HL at frequencies of 6 and 8 kHz. An ANOVA of the average audiometric thresholds for all eight frequencies (0.125–8 kHz) with age group and audiometric frequency as factors showed that the main effects of age group (*F*_1,30_ = 6.192, *p* = 0.009) and audiometric frequency (*F*_7,210_ = 4.104, *p* = 0.020) were significant and that there was a significant interaction between age group and audiometric frequency (*F*_7,210_ = 3.402, *p* = 0.039). These results indicate that the two groups of participants differed in hearing sensitivity. In addition, the interaural difference between the younger and older groups was less than 15 dB (at each frequency). These results indicate that all of the participants had symmetrical hearing between the left and right ears.

All of the participants provided informed consent to join the present study, which was conducted in accordance with the Declaration of Helsinki and was approved by the Ethics Committee of Nanjing Normal University.

#### Experimental Stimuli, Apparatus, and Procedure

The meaningless Chinese target sentence stimuli and masker sentences were the same as those used in Experiment 1. Unlike in Experiment 1, there were three masker-priming conditions: no priming, same-sentence masker-priming, and different-sentence masker-priming. For the same-sentence masker-priming, the priming sentence was presented with the same voices and content as the masker. For the different-sentence masker-priming, the masker-priming sentences were spoken by the masker talker so that the voices were the same for all of the masker-priming and corresponded to that of the masker sentences. However, the contents of the masker-priming sentences were different from those of the masker sentences.

In each experimental trial, each participant pressed a button on a keyboard button to start. In the case of no priming, the speech stimuli were presented according to the following procedure: (1) two-talker masker presentation began immediately after the button press; (2) a full target sentence was presented about 500 ms after the masker onset; (3) the masker and target ended simultaneously. For the same-sentence or different-sentence masker-priming, the speech stimuli were presented according to a four-step process: (1) either the same-sentence or different-sentence masker-priming was presented three times in silence immediately after the button press, and there was no pause between the three presentations; (2) a masker sentence was presented about 4000 ms after the end of the prime presentation; (3) a full target sentence was presented about 500 ms after the masker onset; (4) the masker and target ended simultaneously.

All of the other aspects, including the apparatus and procedure, were the same as in Experiment 1. The performance was scored as the number of correctly identified characters for each keyword. The percentage correct for each keyword was the ratio of the number of characters identified correctly to the total number of characters in each keyword.

### Results

**FIGURE 5:**
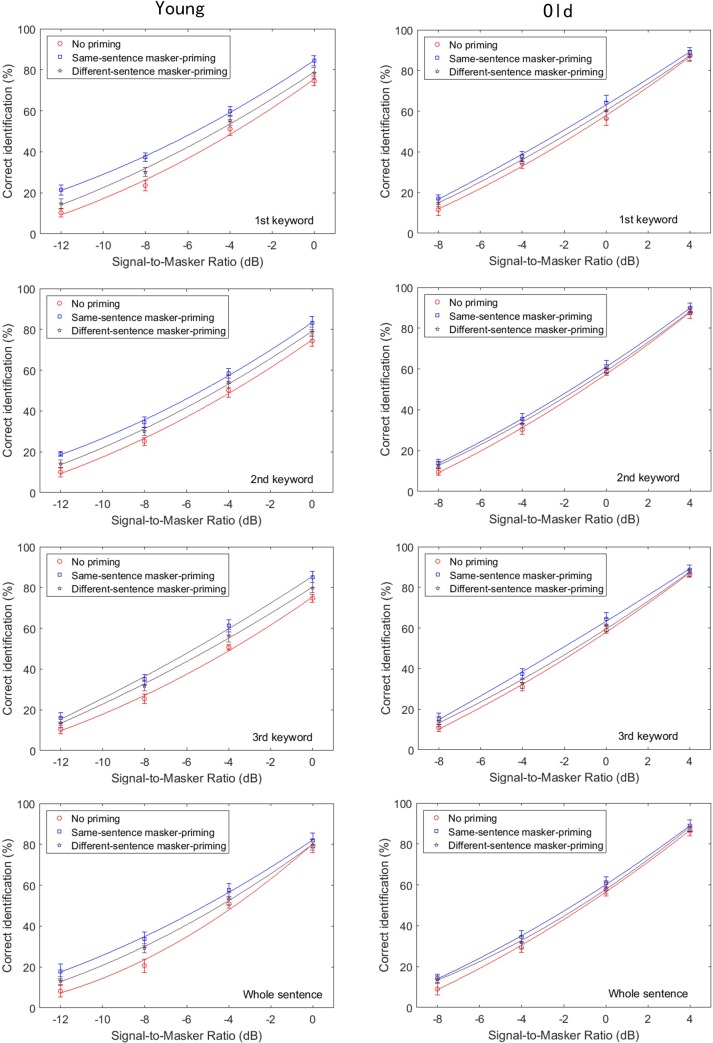
Group-mean percentages of correct identification of each of the three keywords in the target sentence in Experiment 2, as a function of SMR and for the younger **(left)** and older participants **(right)**. The **top, upper middle, lower middle, and bottom panels**, respectively, represent the group-mean percentages of correct identification of the initial keyword, middle keyword, final keyword, and whole target sentence. Two different priming conditions were implemented: no priming (circles) and different-sentence masker-priming (squares). The best-fit psychometric functions (curves) for each masker-priming condition are shown in each of the panels.

**Figure 5** displays the group-mean percentages for correct identification of each keyword (top: initial keyword, upper middle: middle keyword, lower middle: final keyword, bottom: whole sentence) of the target sentences as a function of the SMR for the younger (left) and older participants (right). The results are shown for the cases in which no priming (circles), same-sentence masker-priming (squares), and different-sentence masker-priming (pentagons) was presented. The smooth lines are the best-fit psychometric functions. It is apparent from **Figure 5** (upper six panels) that higher group-mean percentages of correct identification for each of the three keywords were obtained with same-sentence masker-priming than in the other two cases by both the younger and older adults. The bottom panels present the results for correct identification of the whole target sentence. As was the case for each of the three keywords, same-sentence masker-priming yielded a higher percentage of correct identification than did different-sentence masker-priming and no priming. Comparison of the results for the younger and older participants in **Figure 5** indicates that the amount of release from speech masking induced by the same-sentence masker-priming was greater for the younger adults (1.8, 1.5, 2.0, and 1.4 dB for the initial keyword, middle keyword, final keyword, and whole sentence, respectively) than for the older adults (1.1, 0.6, 0.9, and 0.6 dB for the initial keyword, middle keyword, final keyword, and whole sentence, respectively), which agrees with the results of Experiment 1. In addition, **Figure 5** also indicated that the amount of release due to the different-sentence masker-priming was greater for the younger adults (1.0, 0.7, 1.2, and 0.7 dB for the initial keyword, middle keyword, final keyword, and whole sentence, respectively) than for the older adults (0.5, 0.4, 0.4, and 0.2 dB for the initial keyword, middle keyword, final keyword, and whole sentence, respectively).

**FIGURE 6:**
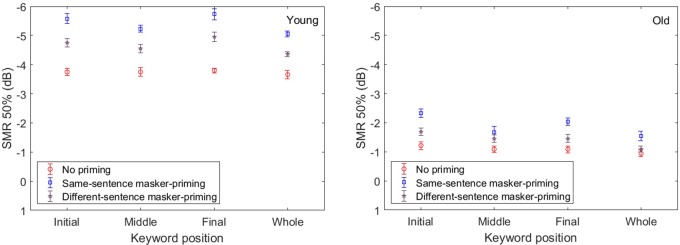
Average μ as a function of keyword position in Experiment 2, for the younger and older participants with and without masker-priming. The blue and red rectangles indicate the data obtained with different-sentence masker-priming and without priming, respectively. The **(left and right)**, respectively, correspond to the average μ for the younger and older participants.

**Figure 6** depicts the changes in the threshold values as functions of keyword position and priming condition for both the younger and older participants. In this figure, the threshold values are lower for the younger adults than for the older adults for each keyword and the whole sentence in all three masker-priming conditions, as was the case in Experiment 1. In addition, the threshold values are lower with same-sentence masker-priming than with different-sentence masker-priming and without priming, and the threshold values are the highest without priming. **Figure 6** indicates that both the younger and older participants could benefit from the voice cues of the masker when identifying the target sentence during the target/masker presentation even when a different sentence was used as the masker prime. Furthermore, as expected, the amount of release from speech masking induced by either same-sentence or different-sentence masker-priming was greater for the younger adults than for the older adults. Finally, with same-sentence masker-priming, the release of the final keyword from the speech masking was the greatest for the younger adults, but the release of the initial keyword from the speech masking was the greatest for the older adults. With different-sentence masker-priming, the amount of release from speech masking was greater for the final keyword than for the other two keywords for the younger adults, but the amount of release from speech masking did not exhibit obvious differences among the three keywords for the older adults.

A three-way ANOVA on the threshold values with age as a between-subjects factor and keyword position and priming condition as within-subject factors showed that there were highly significant effects of age (*F*_1,30_ = 96.585, *p* = 0.000) and priming condition (*F*_2,60_ = 34.223, *p* = 0.000). However, the main effect of keyword position on threshold value was not significant (*F*_2,60_ < 1). In addition, there was no significant interaction between age and priming condition (*F*_2,60_ < 1) or between keyword position and age (*F*_2,60_ < 1). Meanwhile, the interaction between keyword position and priming condition was significant (*F*_2,60_ = 28.683, *p* < 0.02), as was the three-way interaction among age, keyword position, and priming condition (*F*_2,60_ = 31.265, *p* < 0.01).

To confirm the source of the three-way interaction, two-factor ANOVAs with keyword position and priming condition as within-subject factors were conducted separately for the younger and older participants. For the younger participants, the ANOVA confirmed that the main effect of priming condition was significant (*F*_2,30_ = 18.874, *p* < 0.01), but the main effect of keyword position was not (*F*_2,30_ = 1.281, *p* > 0.05). In addition, the ANOVA confirmed that the interaction between keyword position and priming condition was significant (*F*_2,30_ = 14.265, *p* < 0.01). Therefore, the effect of priming condition differed among the three keywords for the younger participants.

For the initial keyword, multiple *t*-tests confirmed that there were significant differences between no priming and same-sentence masker-priming (*t*_15_ = 6.887, *p* < 0.01) and between no priming and different-sentence masker-priming (*t*_15_ = 4.224, *p* < 0.01), but not between same-sentence and different-sentence masker-priming (*t*_15_ = 2.318, *p* > 0.05). In addition, for the middle keyword, the *t*-tests showed that there were significant differences between no priming and same-sentence masker-priming (*t*_15_ = 5.581, *p* < 0.01) and between no priming and different-sentence masker-priming (*t*_15_ = 3.846, *p* < 0.01), but not between same-sentence and different-sentence masker-priming (*t*_15_ = 1.227, *p* > 0.05). However, for the final keyword, the *t*-tests showed that there were significant differences between no priming and same-sentence masker-priming (*t*_15_ = 7.532, *p* < 0.01), between no priming and different-sentence masker-priming (*t*_15_ = 6.446, *p* < 0.01), and between same-sentence and different-sentence masker-priming (*t*_15_ = 4.854, *p* < 0.01). For the difference between no priming and same-sentence masker-priming, the multiple *t*-tests also confirmed that the release of the initial keyword was not significantly different from that of the middle keyword (*t*_15_ = -1.616, *p* > 0.05), but the release of the final keyword was greater than that of the initial (*t*_15_ = -3.288, *p* < 0.01) and middle keywords (*t*_15_ = -4.152, *p* < 0.01). For the difference between no priming and different-sentence masker-priming, the multiple *t*-tests also showed that the release of the initial keyword was not significantly different from that of the middle keyword (*t*_15_ = -1.092, *p* > 0.05), but the release of the final keyword was greater than that of the initial (*t*_15_ = -2.653, *p* < 0.05) and middle keywords (*t*_15_ = -3.679, *p* < 0.01). For the difference between same-sentence and different-sentence masker-priming, the *t*-tests showed that the release of the initial keyword was not significantly different from that of the middle keyword (*t*_15_ = -1.139, *p* > 0.05), but the release of the final keyword was greater than that of the initial (*t*_15_ = -3.107, *p* < 0.01) and middle keywords (*t*_15_ = -3.741, *p* < 0.01). Therefore, both same-sentence and different-sentence masker-priming could effectively enable the release of the keywords from the masking, same-sentence masker-priming induced a greater release than different-sentence masker-priming, and the amount of release from the masking due to masker-priming was greater for the final keyword than for the other two keywords among the younger participants.

For the older participants, an equivalent two-factor ANOVAs with keyword position and priming condition as within-subject factors confirmed that priming condition had a significant main effect (*F*_2,30_ = 15.668, *p* < 0.01), but keyword position did not (*F*_2,30_ < 1). In addition, the ANOVA confirmed that the interaction between keyword position and priming condition was significant (*F*_2,30_ = 10.904, *p* < 0.01). Hence, the effect of priming condition differed among the three keywords for the older adults.

The multiple *t*-tests confirmed that, for the initial keyword, there were significant differences between no priming and same-sentence masker-priming (*t*_15_ = 5.406, *p* < 0.01) and between no priming and different-sentence masker-priming (*t*_15_ = 3.957, *p* < 0.01), as well as between same-sentence and different-sentence masker-priming (*t*_15_ = 3.225, *p* < 0.05). For the middle keyword, the *t*-tests showed that there were significant differences between no priming and same-sentence masker-priming (*t*_15_ = 4.940, *p* < 0.01) and between no priming and different-sentence masker-priming (*t*_15_ = 3.913, *p* < 0.01), but not between same-sentence and different-sentence masker-priming (*t*_15_ = 1.543, *p* > 0.05). Meanwhile, for the final keyword, the *t*-tests showed that there were significant differences between no priming and same-sentence masker-priming (*t*_15_ = 4.725, *p* < 0.01) and between no priming and different-sentence masker-priming (*t*_15_ = 3.761, *p* < 0.01), but not between same-sentence and different-sentence masker-priming (*t*_15_ = 1.031, *p* > 0.05). For the difference between no priming and same-sentence masker-priming, the multiple *t*-tests also confirmed that the release of the initial keyword was greater than that of the middle (*t*_15_ = -3.957, *p* < 0.05) and final keywords (*t*_15_ = -2.829, *p* < 0.01), but the release of the middle keyword was not significantly different from that of the final keyword (*t*_15_ = -1.124, *p* > 0.05). For the difference between no priming and different-sentence masker-priming, the multiple *t*-tests again showed that the release of the initial keyword was greater than that of the middle (*t*_15_ = -3.773, *p* < 0.01) and final keywords (*t*_15_ = -3.091, *p* < 0.01), but the release of the middle keyword was the not significantly different from that of the final keyword (*t*_15_ = -1.181, *p* > 0.05). For the difference between same-sentence and different-sentence masker-priming, the *t*-tests showed that the release of the initial keyword was greater than that of the middle (*t*_15_ = -3.224, *p* < 0.05) and final keywords (*t*_15_ = -3.872, *p* < 0.01), but the release of the middle keyword was not significantly different from that of the final keyword (*t*_15_ = -1.056, *p* > 0.05). Therefore, for the older adults, either same-sentence or different-sentence masker-priming could effectively lead to release from the masking and same-sentence masker-priming induced a greater release than different-sentence masker-priming, as was the case for the younger adults. However, the amount of release from masking due to masker-priming was greater for the initial keyword than for the other two keywords among the younger participants.

For the whole target sentences, a two-factor ANOVA showed that the main effects of both age (*F*_1,30_ = 98.085, *p* = 0.000) and priming condition (*F*_2,60_ = 26.986, *p* = 0.000) were significant, but the interaction between age and priming condition was not significant (*F*_2,60_ < 1). The multiple *t*-tests showed that all three priming conditions differed from each other for the whole target sentence (no priming vs. same-sentence masker priming, *t*_15_ = 4.317, *p* < 0.01; no priming vs. different-sentence masker priming, *t*_15_ = 3.646, *p* < 0.01; same-sentence masker priming vs. different-sentence masker priming, *t*_15_ = 2.919, *p* < 0.05). Therefore, the thresholds were higher for the older participants than for the younger participants, and the thresholds were the highest without priming, which implies that masker-priming leads to release from speech masking. In addition, the amount of release from speech masking was greater with same-sentence masker-priming than with different-sentence masker-priming for the identification of the whole target sentence.

### Discussion

In Experiment 2, there were three masker-priming types: no priming, same-sentence masker-priming, and different-sentence masker-priming. For the same-sentence masker-priming, the priming sentence was presented with the same voices and content as the masker. For the different-sentence masker-priming, the masker-priming sentences were spoken by the masker talkers so that the voices were the same as the masker. However, the content of the masker-priming sentence differed from that of the masker sentence. We examined whether familiarity with the voices of the masker talkers alone could improve the target identification performance and generate release from speech masking when the target and masker speech were presented. We also examined whether there were differences between the release from speech masking under the two kinds of masker-priming conditions. The results showed that both same-sentence masker-priming (voice and content cues) and different-sentence masker-priming (voice cue alone) could effectively lead to release from the masking in both age groups. There was a very small but significant release from speech masking when the different masker-priming sentences were presented before the target/masker presentation for both the younger and older participants. In other words, providing listeners with voice information of the masker oriented their attention to the targets in the target/masker complexes. With different-sentence masker-priming, because the contents of the primes were unrelated to the masker sentences, the effect of the content was minimized. Providing the content and voice information of the masker (the same-sentence masker-priming condition) also improved the target identification performance, it is reasonable to conclude that the benefits of masker-priming in the present study were due to both the voice and content of the masker. Like the results of Experiment 1, those of Experiment 2 show that the amount of release from speech masking induced by either same-sentence or different-sentence masker-priming was greater for the younger participants than for the older participants. Furthermore, the results of Experiment 2 revealed classical serial position effects (Deese and Kaufman, 1957; Murdock, 1962) on the masking release due to both same-sentence and different-sentence masker-priming among both the younger and older listeners. Among the younger participants, the amount of the masking release was greater for the final keyword than for the other two keywords, which is consistent with the recency effect. Among the older participants, there was a primacy effect, i.e., the release from masking was greater for the initial keyword than for the other two keywords.

## General Discussion

The purpose of this study was to examine whether listeners can use prior information about a masker to improve word recognition in speech masking and whether there are age-related differences in the effectiveness of masker cues for release from speech masking between older and younger adults.

In Experiment 1, both the younger and older participants were presented with the same complete sentence as a two-talker masker immediately before presenting the masker/target. The masker primes with the same masker sentence led to release from the two-talker masker in both age groups, but the amount of release from the masker was greater for the younger listeners than for the older listeners. Thus, the present results demonstrate that this masker-priming produces a release from informational masking and that there are age-related differences between younger and older listeners.

In Experiment 2, there were three masker-priming conditions: no priming, same-sentence masker-priming, and different-sentence masker-priming. Same-sentence masker-priming provided the listeners with the voice and content cues of the masker simultaneously, while different-sentence masker-priming provided the listeners with the voice cue of the masker talker alone. We examined whether there were differences in the release from speech masking between the two kinds of masker-priming conditions. The present results show that both same-sentence and different-sentence masker-priming could effectively lead to release from masking in both age groups. There was a very small but significant release from speech masking when the different masker-priming sentences were presented in silence before the target/masker presentation among both the younger and older participants. One possible interpretation of the different-sentence masker-priming effect is that providing knowledge about the vocal characteristics of the masker talker was beneficial in parsing an auditory scene because it helped the listeners orient their attention to the targets in the target/masker complex. However, for both kinds of primes, there was greater release from the two-talker masker among the younger listeners than among the older listeners. Thus, it can be concluded that familiarity with the voice alone could also produce a release from information masking that was not equivalent for the two age groups. Taken together, the present results indicate that both younger and older listeners benefit from voice and content in cueing masker speech when attempting to recognize speech in a competing speech scene. In addition, the release from the masker due to the voice cue alone was less than that due to the combined voice and content cues in both age groups.

The masker-priming effect might result from a repetitive inhibitory effect of the human central auditory system (Ulanovsky et al., 2003; Perez-Gonzalez et al., 2005; Grill-Spector et al., 2006) due to both the voice and content of the masker during the target/masker presentation. The repetitive inhibitory effect leads to neural activity reduction. Providing the listeners with masker information (voice or/and content) of the masker oriented their attention to the targets among the target/masker complexes, leading to the release of the target speech from the familiar masker. The decreased release from the two-talker masker among the older listeners suggests an age-related decline of the masker-priming effect. The present study revealed a small but significant release from masking among the older listeners. This age-related decline in the repetitive inhibitory effect for the older listeners could be due to decreased adaptation of the central auditory systems in their brains in response to repeated interference control. Although masker primes familiarize listeners with masker information, thereby orienting their attention to the targets against speech masking, decreased adaptation of their central auditory systems made it more difficult for the older listeners to follow the target speech in the target/masker complex. The decreased release from the two-talker masker among the older listeners could also be due to age-related audiometric deficits, preventing the older listeners from benefiting from the masker-priming during the target/masker presentation. In other words, the lesser amount of masking release among the older participants may have resulted from reduced audiometric sensitivity associated with high-frequency hearing loss and aging. It has been shown that age-related audiometric deficits in speech perception (Moore, 2007; Hopkins and Moore, 2011) are related to high-frequency hearing loss and have deleterious influences on speech perception even for very small hearing losses (30 dB or less).

These results of this study seem to be consistent with those of auditory enhancement effect studies (Viemeister, 1980; Viemeister and Bacon, 1982; Byrne et al., 2011), which indicated that providing listeners with a homophonic sequence with a certain pure tone deleted before presenting the full homophonic sequence with this pure tone could facilitate the following of this pure tone within the homophonic sequence. In other words, the masker cue (the homophonic sequence without a certain pure tone) could orient the attention of the listeners to the emerging target (pure tone) during the presentation of the target/masker complex (complete homophonic sequence). In fact, listeners are sensitive to emerging sounds if they are exposed to a noisy environment for a certain time. In this case, it is possible that familiarity with the masking environment helps listeners perceive target speech. Exposure to the masking environment familiarizes listeners with the masker and leads to central auditory system inhibition and decreased neural activity. The target speech then arouses the auditory nerve system and orients the attention of the listeners to the targets, leading to the release of the target speech from the familiar masker.

However, the present results are inconsistent with the findings obtained by Zhang et al. (2012), who actually found that a single exposure to a masker prime in silence increased the informational masking in a competing speech environment when the interval between the masker prime and target/masker presentation was short. They speculated whether the increasing masking effect originated from insufficient exposure to the masker prime and the short delay between the masker prime and the speech presented in the masker. In fact, the exposure time and interval between the masker prime and target/masker presentation played important roles in masker release in this study. As mentioned above, the preliminary experiment before the formal experiment confirmed that more repetitions of the masker priming and longer delay could lead to the greater masking release. It is highly likely that these factors affect the repetitive inhibitory effect of the auditory central system and thereby the effectiveness of masker primes in facilitating target speech identification. It is expected that further investigation of how these factors affect the repetitive inhibitory effect will be necessary. In addition, another likely explanation for the discrepancy between the present results and those reported by Zhang et al. (2012) could be differences between the scoring methods. In the report of Zhang et al. (2012), the percentage correct was the number of whole words correctly identified divided by the total number of target words presented. In the present study, the percentage correct for a word was the number of characters (syllables) identified correctly divided by the total number of characters.

Before concluding, we note that the present study also found that the masking release induced by masker-priming was different among three keywords in both age groups. For the younger participants, the masking release was greatest by far for the final keyword. For older participants, the masking release for the initial keyword was greater than the other two keywords. These positional effects appear to be consistent with the classical serial position effects (Deese and Kaufman, 1957; Murdock, 1962).

## Conclusion

The present study focused on whether familiarity with masker speech can improve target speech identification in the presence masker speech by younger and older listeners. The age-related differences of the masker cuing effect on the release of speech from informational masking were also studied. The results indicate that although the masker-priming effect declines in older adults, masker-priming can improve target sentence recognition for both younger and older adults. Furthermore, the identification of target speech with same-sentence masker-priming depended significantly upon the positions of the three keywords, for both the younger and older adults. However, with different-sentence masker-priming, the position-dependent effect was significant only for the younger participants, but not for the older participants. The present results suggest that, regardless of the target speech cues, masker speech cues may also be utilized to release targets from maskers in noisy environments.

## Data Availability Statement

All of the relevant data for this study are included within the manuscript.

## Ethics Statement

The participants gave their written informed consent to participate in the experiment, and were paid a modest stipend for their participation.

## Author Contributions

TF, QC, and ZX contributed the study concept and design. TF organized the database and wrote the first draft of the manuscript. QC performed the statistical analysis. TF, QC, and ZX wrote parts of the manuscript. All the authors have read and approve of this version of the manuscript, ensuring the integrity of the work.

## Conflict of Interest Statement

The authors declare that the research was conducted in the absence of any commercial or financial relationships that could be construed as a potential conflict of interest.
